# Integrating Physiatry and Palliative Care in Outpatient Oncology: A Clinical Framework for Bidirectional Referral and Co-Management

**DOI:** 10.3390/curroncol33070387

**Published:** 2026-06-25

**Authors:** Emmanuel G. Villalpando, Jamie Fertal, Finly Zachariah, Jeannine M. Brant, Jessica T. Cheng

**Affiliations:** 1 OPTI-West Physical Medicine and Rehabilitation Program, Casa Colina Hospital and Centers for Healthcare, Pomona, CA 91767, USA; egvillal16@gmail.com; 2 Division of Supportive Medicine, Department of Supportive Care Medicine, City of Hope Orange County, Irvine, CA 92618, USA; jfertal@coh.org; 3 Division of Supportive Medicine, Department of Supportive Care Medicine, City of Hope Comprehensive Cancer Center, Duarte, CA 91010, USA; fzachariah@coh.org; 4 Clinical Nursing Science & Innovation, City of Hope Comprehensive Cancer Center, Duarte, CA 91010, USA; jbrant@coh.org; 5 Department of Supportive Care Medicine, City of Hope Orange County, Irvine, CA 92618, USA

**Keywords:** physical and rehabilitation medicine, palliative care, neoplasms, cancer survivors, referral and consultation, patient care team, implementation science, functional status

## Abstract

Patients with cancer often experience intertwined symptom burden and functional decline that contribute to falls, unsafe transfers, uncontrolled symptoms, caregiver strain, and crisis-driven care. Physical medicine and rehabilitation (PM&R), also known as physiatry, and specialty PC both address suffering and quality of life through complementary clinical approaches; however, collaborative care with and between these two specialties is inconsistent in routine oncology practice. This paper presents a clinical implementation framework informed by targeted literature synthesis for bidirectional referral and co-management between PM&R and PC in oncology. The framework was informed by the PC referral criteria literature, cancer rehabilitation triage literature, trigger-based serious illness identification models, and implementation science. Four clinic-usable tools are proposed, including a scope and overlap map, a clinical-needs gradient, a referral trigger table linking common clinical signals to the reason for referral and expected clinical actions, and a primary-service triage workflow. This framework is intended to clarify which service is best positioned to be the primary supportive service according to the patient’s current needs, when rehabilitation therapy alone may be sufficient, and when co-management should be the default. This concept-to-practice model is designed to facilitate early, needs-based referrals and coordinated supportive care in oncology settings.

## Simple Summary

Patients with cancer often experience both complex symptoms and functional decline, but it is not always clear when physiatry, palliative care (PC), or rehabilitation therapy should be involved alone or in collaboration. This paper presents a practical clinical framework to guide those decisions. It defines the distinct and overlapping roles of physiatry and PC through four clinical tools—a scope and overlap map, a clinical-needs gradient, a referral trigger table linking rationale and management expectations, and a proposed triage workflow for determining which service should be the primary supportive service according to the patient’s current needs. The goal is to support earlier, needs-based referral and consistent care collaboration in oncology practice.

## 1. Introduction

Cancer often leads to a predictable clinical cascade depending on the stage of disease. In advanced or recurrent cancer, as symptoms and treatment toxicities worsen, activity declines, and deconditioning accelerates. These symptoms and impairments often accumulate cyclically. As function worsens, patients become more dependent on caregivers and more vulnerable to falls, unsafe transfers, equipment delays, medication-related instability, and emergency care. These problems rarely occur in isolation. Instead, symptom burden, functional decline, and caregiver strain often reinforce one another and contribute to late crises that undermine quality of life and disrupt care [1,2].

Two complementary specialty disciplines are especially well positioned to mitigate morbidity and reduce suffering from this cascade, specifically physical medicine and rehabilitation (PM&R) and specialty palliative care (PC). PM&R physicians, or physiatrists, complete medical school followed by 4 years of residency. Some complete a fellowship year in the rapidly growing subspecialty of cancer rehabilitation medicine. PM&R, as the medical specialty of function, addresses physical and cognitive impairments through diagnostic clarification, mobility and falls assessment, multimodal rehabilitation prescription, equipment prescription, and procedures for selected pain or spasticity syndromes [3,4]. PM&R prescribes rehabilitation therapy and closely collaborates with physical therapy (PT), occupational therapy (OT), and speech-language pathologists (SLP), among other rehabilitation team members, but are not rehabilitation therapists themselves. They are trained to lead rehabilitation teams in the outpatient, hospital, and inpatient rehabilitation settings. As such, PM&R physicians are positioned to bridge the gap between oncologists, medical specialties, and others, with the rehabilitation team, providing unique opportunities to provide comprehensive rehabilitation across impairments, across functional abilities, across time, and across healthcare settings [5].

Specialty PC addresses multidimensional suffering through complex symptom management, communication about goals and prognosis, psychosocial and spiritual support, and care transitions across the trajectory of serious illness in a patient- and family-centered approach [6,7]. This multidimensional care is often accomplished with teams involving nursing, social work, chaplaincy, mental health specialists, and possibly rehabilitation therapists. Both PM&R and PC aim to improve quality of life through complementary mechanisms.

Preliminary evidence and limited literature support the value of rehabilitation in advanced cancer and palliative settings. In the Pal-Rehab trial (n = 288), patients with newly diagnosed advanced cancer were randomized to a 12-week palliative rehabilitation program consisting of mandatory consultations with a specialized PC team (physician, nurse) and access to an interdisciplinary group program including PT-led exercise sessions, OT, and psychosocial support, or to standard oncology care alone. Quality of life improved at 12 weeks in the intervention group [8]. In another trial, 60 patients with advanced cancer receiving inpatient PC were randomized to 30 min physiotherapy sessions of active exercises, myofascial release, and proprioceptive neuromuscular facilitation three times weekly for two weeks, or to no exercise. Fatigue was significantly reduced in the treatment group [9]. Observational and mixed-method studies have further suggested that palliative rehabilitation is feasible, acceptable, and associated with gains in mobility, symptom relief, and satisfaction [10,11,12]. More broadly, the cancer rehabilitation literature has shown that functional impairments are common, undertreated, and often manageable when identified systematically [3,13,14]. Collectively, these studies have shown potential benefits of limited non-comprehensive rehabilitative care in advanced cancer, and the conceptual importance of rehabilitative care integration into oncologic care.

Although conceptual overlap between cancer rehabilitation and PC has been established [2], consistent integration into oncology care is challenged. The importance of integrating PM&R and rehabilitation services into cancer care has been recognized by the Commission on Cancer standards [15] and numerous oncology guidelines [16]. However, there remains a wide gap in both guideline inclusion [17] and utilization relative to the high prevalence of functional impairments in cancer survivors [13], revealing a need for clinical operational guidance. Several lines of related work have addressed components of this challenge. On the rehabilitation side, oncology rehabilitation triage models have shown feasibility while also highlighting referral uncertainty as a common barrier to routine referrals in clinical practice [14]. On the PC side, needs-based referral criteria for outpatient specialty PC have been established through international consensus [18,19]. To address referral uncertainty, structured trigger systems in serious illness care have shown that workflow prompts embedded into the electronic health record (EHR) can support consistent recognition of supportive care needs. However, these lines of work have not yet been integrated into a single clinical framework for bidirectional referral and co-management between PM&R and PC.

The subsequent challenge is to translate this conceptual overlap between rehabilitation and PC into a practical, repeatable, and coordinated clinical workflow with PM&R and PC. In busy oncology settings, clinicians may recognize complex symptom burden, functional decline, or caregiver strain without having a clear way to decide which specialty service should be the primary supportive service, when rehabilitation therapy alone may be sufficient, and when co-management should be the default. This paper presents a clinical implementation framework informed by targeted literature synthesis for bidirectional referral and co-management between PM&R and PC in cancer. It is organized around four tools, a scope and overlap map, a clinical-needs gradient, a referral trigger table, and a primary-service triage workflow designed to support consistent, needs-based referral in clinical practice.

## 2. Methods

This paper presents a clinical framework developed through a targeted literature review and iterative refinement through a small collaborative PM&R and PC practice within an NCI-designated Comprehensive Cancer Center network site. The targeted literature review included three bodies of literature. The first was cancer rehabilitation triage and impairment-routing literature, including models designed to distinguish patients who require PM&R physician involvement from those appropriate for rehabilitation therapy alone [3,4,5,14,20]. The second was PC referral criteria literature, including needs-based outpatient referral models and consensus-derived trigger approaches [18,19]. The third was literature on screening-based identification of supportive care needs in serious illness in intensive care and other high-acuity settings, where standardized clinical prompts have been used to reduce variability in the recognition of supportive care needs [21,22].

The search strategy included five databases (PubMed/MEDLINE, Embase, CINAHL, the Cochrane Library, and Google Scholar). Search terms included physiatry, rehabilitation, palliative care, trigger, referral, and triage. Additional citations were found by citation tracking, hand-searching relevant clinical practice guidelines and standards, and references recommended by co-authors. The search was conducted iteratively between September 2025 and April 2026. The literature reviewed consisted of English-language, peer-reviewed publications or guidelines and standards from recognized professional organizations that addressed oncology, rehabilitation, or palliative care populations. Foundational works regardless of date published and literature from the preceding three years were emphasized upon review.

The framework was developed and refined through a multi-stage, iterative process involving PM&R and PC clinicians. In the first stage, stakeholder-informed discussions with clinicians with and without prior collaboration experience identified a gap in structured approaches to PM&R-PC clinical collaboration in oncology care settings. Initial tool drafts were developed from both clinical experience and literature synthesis, with each informing subsequent refinement. In the second stage, the tools underwent expert review by clinicians experienced in PM&R-PC collaboration, assessing clinical accuracy, terminology, and workflow alignment across specialties. This was followed by additional review by clinicians without prior or with limited prior collaboration experience and a clinical researcher, assessing clarity and usability. In the final stage, the tools were applied in clinical practice by a clinician without prior PM&R-PC collaboration experience to assess usability and interpretability in a real-world oncology setting. Revisions were made based on feedback.

Clinical vignettes were developed as composite cases to illustrate application of the framework across a range of supportive care needs. Vignettes were informed by clinical experience and refined through iterative expert review to ensure clinical relevance and clarity. All vignettes are hypothetical and intended for illustrative purposes.

## 3. Clinical Tools

Four clinical tools were developed detailing distinctions and synergies between PM&R and PC for the purpose of facilitating triage and bidirectional referrals. Clinical vignettes were developed to illustrate distinctive and synergistic dynamic supportive oncology care collaboration.

### 3.1. Scope and Overlap Map (Figure 1)

The scope and overlap map was designed and drafted from clinical experience, then refined by literature review and iterative expert review to map the distinct and shared clinical domains of PM&R and PC [3,4,5,14,20]. The PM&R domain includes the core PM&R scope, with cancer-related conditions, populations, and interventions added only when not already conceptually included. For example, aromatase inhibitor-associated musculoskeletal syndrome is conceptually included in musculoskeletal diagnosis and procedures. Chemotherapy-induced peripheral neuropathy is conceptually included in neurologic workup, falls, and equipment.

The unifying core of the PM&R specialty is the optimization of functional ability, primarily in neuromuscular conditions. Core competencies of PM&R include the evaluation of impairment and functional limitations across multiple domains, prescription of durable medical equipment, and coordination of multidisciplinary therapies [23]. These uniquely position the specialty to address complex rehabilitation needs of cancer survivors from prehabilitation through long-term survivorship. PM&R physicians often subspecialize in interventional pain, pain medicine, spine medicine, sports medicine, brain injury, spinal cord injury, stroke, neuromuscular disorders, electrodiagnostic medicine, spasticity, pediatrics, burn, limb loss, cancer rehabilitation medicine, or palliative care. Further specialization areas include regenerative medicine, musculoskeletal ultrasound, lifestyle medicine, integrative medicine, cardiopulmonary rehabilitation, advanced technologies such as robotics and wearable sensors, adaptive sports, gaming rehabilitation, and pelvic floor rehabilitation. The broad scope of the PM&R field leads to variation in clinical services and procedures provided by each physician.

**Figure 1:**
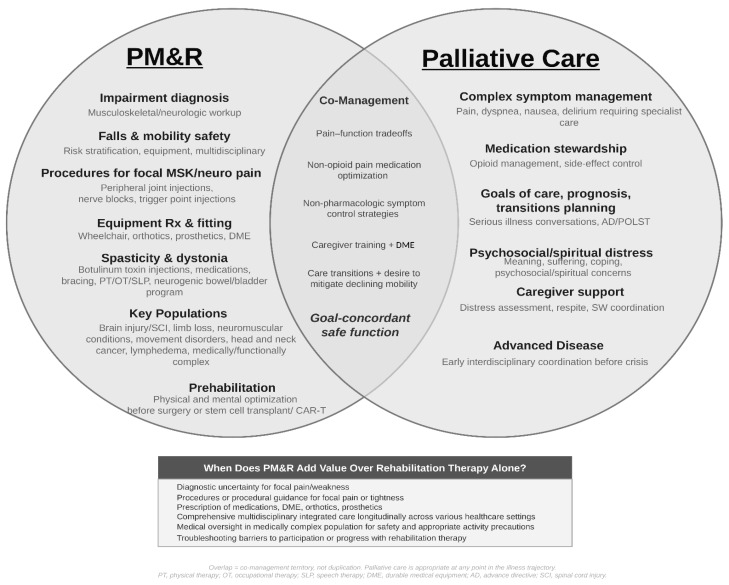
Scope and overlap map of PM&R and PC in cancer. Each discipline has distinct clinical territory, with a shared co-management zone where symptoms and function are tightly interdependent. The lower panel distinguishes when PM&R physician involvement adds value beyond rehabilitation therapy alone. PM&R, physical medicine and rehabilitation; MSK, musculoskeletal; neuro, neurological; Rx, prescription; DME, durable medical equipment; PT, physical therapy; OT, occupational therapy; SLP, speech-language pathology; SCI, spinal cord injury; CAR-T, chimeric antigen receptor T-cell therapy; AD, advanced directives; POLST, physician orders for life-sustaining treatment; SW, social work.

The specialty fields of PM&R and PC commonly have limited interaction in clinical practice. In such settings, scope distinctions and overlaps may be relatively clear, as depicted in Figure 1. PM&R is generally the best primary supportive service when the dominant need is impairment-driven functional decline that requires diagnostic clarification, medical oversight, targeted rehabilitation planning, or higher-risk safety management. In cancer, this may include focal weakness, falls or near-falls, mobility decline, focal musculoskeletal or neurologic pain, spasticity, lymphedema, neurogenic bowel or bladder dysfunction, dysphagia, and equipment needs that require documentation, prescription, or fitting [3,4]. PM&R adds value when the clinical problem extends beyond a single course of a single rehabilitation therapy discipline and requires physician-level assessment or intervention. Cancer prehabilitation through PM&R, which involves multimodal and multidisciplinary interventions to optimize physical and mental abilities before cancer surgery, stem cell transplant, or CAR-T cell therapy, is a rapid area of growth where the PM&R specialty is uniquely positioned to optimize performance status and mitigate cancer-related and cancer treatment-related morbidity [24,25,26,27].

PC is generally the best primary supportive service when the dominant need is multidimensional suffering that requires specialist support. This includes complex symptoms requiring specialist care, goals-of-care discussion or prognostic clarification, psychosocial or spiritual distress, caregiver distress, and care transitions across settings [6,7]. PC also commonly manages medication-related tradeoffs that can affect both symptoms and function, including sedation, constipation, delirium risk, and polypharmacy-related instability [6,7,28,29].

The overlap between PM&R and PC is common and clinically important. This is the space in which symptoms and function are tightly interdependent and neither problem can be managed well in isolation. Examples include pain that limits mobility, medication changes that affect balance or cognition, caregiver strain related to transfers or equipment needs, and care transitions in which symptom control and functional safety must be addressed together. In this shared space, the central aim is goal-concordant safe function.

Clarification of when to involve PM&R versus rehabilitation therapy alone is also included. For straightforward conditioning, gait training, or ADL retraining without diagnostic uncertainty, red flags, or medical complexity, direct referral to physical therapy, occupational therapy, or speech-language pathology may be sufficient. PM&R physician involvement adds value when the clinical picture involves diagnostic uncertainty, procedural considerations, equipment prescription, medical complexity that requires oversight, or barriers that rehabilitation therapy alone has not been able to resolve.

### 3.2. Clinical-Needs Gradient (Figure 2)

The clinical-needs gradient was designed from clinical experience to spatially represent a patient’s comprehensive supportive clinical needs. Upon review of the literature, similar but different gradients temporally representing increasing palliative care needs with advancing cancer were found, such as in Hawley et al.’s bow tie model [30]. This tool was iteratively revised through cross-specialty expert review by clinicians with varying collaborative experience.

**Figure 2:**
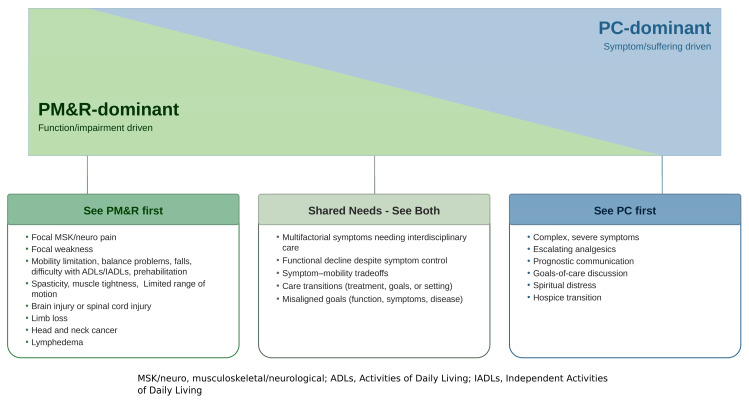
Complementary clinical-needs gradients spatially represent PM&R and PC involvement with five categories ranging from PM&R-specific needs that are function/impairment-driven through shared needs, to PC-specific needs that are symptom/suffering-driven. In the shared needs category, symptoms and function are tightly interdependent.

Cancer rehabilitation medicine is an emerging subspecialty within PM&R in which physiatrists address functional impairments, symptom burden, and disability across the cancer continuum—from diagnosis through survivorship or end of life. As illustrated in Figure 2, cancer rehabilitation medicine and PC are complementary function- and quality-of-life-directed specialties within oncology care.

Most patients with advanced cancer do not fall neatly into a single service lane. Instead, their dominant supportive clinical needs range from primarily function and impairment-driven problems on one end to primarily symptom and suffering-driven problems on the other, with a broad zone of shared needs in between where symptoms and function are tightly interdependent and the shared goal is goal-concordant safe function. A patient’s supportive oncology clinical needs are often dynamic, fluctuating between categories over time. How these clinical needs are addressed can vary depending on the individualized skillsets of the clinicians involved, access to specialty services, established professional relationships, and patient bandwidth to engage in medical care.

There are limitations to this conceptual spatial representation of clinical needs. One could argue, with caveats, that there is a temporality component in this clinical-needs gradient both according to disease stage and time course. PM&R often aligns with earlier-stage disease, addressing functional optimization before cancer treatment with prehabilitation, or after treatment with rehabilitation. In advanced disease stages, distinctions between these specialized supportive oncology needs are less clear. PC is recognized as important and increasingly central as disease progresses further. As disease progresses, so do symptoms and functional impairments. The absolute functional needs commonly rise along the disease progression trajectory. Thus, the PC-dominant category warrants clarification as depicting a lower relative clinical need for outpatient PM&R specifically rather than lower absolute functional needs or decreased benefit of rehabilitative interventions. In the setting of escalating medical needs limiting ability to participate in outpatient care, engagement with rehabilitative multimodal and multidisciplinary interventions led by outpatient PM&R is often challenged though still valuable. Thus, PC clinicians are often the primary supportive clinicians needing to address functional needs with limited PM&R contributions for this patient population with progressing disease, consistent with clinical experience.

Spatial rather than time- or disease progression-based representation of clinical needs was chosen due to caveats further illustrated in the following patient scenarios that do not fit neatly within a temporal model, reflecting the dynamic interrelatedness of disease, function, and symptoms. For example, patients with recently diagnosed advanced gynecological cancer undergoing neoadjuvant chemotherapy may have significant interdependent needs for both PM&R (for prehabilitation and pre-existing musculoskeletal issues limiting mobility) and specialized PC (for symptom management in advanced illness). Patients with newly diagnosed aggressive cancers could rapidly decline in alertness, cognition, and physical function, precipitating a rapid transition in care goals from prehabilitation to hospice. These caveats reveal the importance of cross-disciplinary training between PM&R and PC in oncologic care. They also emphasize that the composite supportive clinical needs are not easily delineated by time course or disease stage, but by itemizing the clinical needs that could benefit from repeat and longitudinal clinical assessment for needs-based and bidirectional referrals to PM&R and PC.

### 3.3. Referral Trigger Table (Table 1)

This table is the operational bridge between screening and action, mapped from a literature review followed by an iterative expert review [3,13,18,19]. It organizes referral triggers into three main categories that reflect PM&R, shared needs, and PC, corresponding conceptually to Figure 2. For each trigger, the table explains the clinical rationale and expectations for evaluation and management, providing referring clinicians with a framework for communicating expectations to patients. The goal is to reduce a common barrier to referral and clinical collaboration, namely recognizing that a problem exists then what the corresponding service(s) is expected to do.

**Table 1:** Referral triggers at a glance: what prompts a referral, why, and what each service does. Each trigger is mapped to why it warrants referral and to what physical medicine & rehabilitation (PM&R) and palliative care (PC) actually deliver.

Referral Trigger	Why Refer	What the Service Does
PM&R-dominant—see PM&R first function/impairment driven		
**Focal MSK/neuro pain or weakness**	Localized, rehab-treatable impairment that needs targeted diagnosis, procedures, and interdisciplinary interventions	Focal workup including EMG/nerve conduction studies; peripheral nerve, joint, and muscle injections
**Spasticity, muscle tightness, limited ROM**	Abnormal tone restricts function and responds to rehab-specific interventions	Spasticity/dystonia care includes botulinum toxin, medications, bracing and equipment Rx coordinated with PT/OT
**Mobility/ADL/IADL limits** **Balance problems, falls** **Prehabilitation**	Functional independence is at risk, the core domain of rehabilitation. Poor performance status risks poor treatment tolerance.	Prescribes tailored PT/OT, equipment: strengthening, gait and transfer training, balance training, neuropathy-focused modalities, ADL/IADL retraining
**Special populations: brain injury; spinal cord injury; limb loss; head and neck cancer**	Specialized multidisciplinary rehab management restores control and prevents complications	Neurogenic bowel and bladder program, spasticity management, equipment Rx and positioning, prosthetic care
**Lymphedema**	Longitudinal collaborative care with lymphedema therapist to prevent complications and address refractory cases	Differential workup, lymphedema therapy and garment Rx
Shared spectrum—see both, co-manage shared needs		
**Symptom–function tradeoffs; functional decline despite symptom control; multifactorial symptoms**	Gains in one domain can cost the other; clinical needs span both teams	PC manages symptoms; PM&R preserves safe function, identifies procedural and rehab interventions for symptom control; tradeoffs weighed jointly
**Care transitions; misaligned goals**	Overlapping needs exceed any single specialty; coordination prevents crises	Shared goal-setting (disease, symptoms, function), an aligned care plan, and coordinated transitions of care
PC-dominant—see PC first symptom/suffering driven		
**Complex, severe or escalating symptoms**	Refractory symptom burden needs multimodal, interdisciplinary management	Pharmacologic and non-pharmacologic symptom optimization; analgesia escalation; management of intended and unintended treatment effects
**Goals-of-care & prognostic communication**	Serious-illness decisions need skilled, prognosis-informed conversations	Goals-of-care discussions; prognostic communication; advance care planning
**Spiritual distress; hospice transition**	Suffering spans biopsychosocial and spiritual domains near the end of life	Spiritual and psychosocial support; hospice transition and end-of-life care
**Why refer to the physician specialty?** PM&R and palliative care are physician specialties. PM&R physicians diagnose, perform rehab-specific procedures, and direct the therapy team; PC physicians lead complex symptom management and goals-of-care. Refer here, not only to therapists or single-discipline services that address similar issues, when the problem needs physician-level diagnosis, medical management, or procedures.		

MSK/neuro, musculoskeletal/neurological; rehab, rehabilitation; EMG, electromyography; ROM, range of motion, Rx, prescription; PT, physical therapy; OT, occupational therapy; ADLs/IADLs, (Independent) Activities of Daily Living.

Table 1 shows the referral trigger table linking common clinical signals to their clinical significance and to expected clinical actions. Triggers are organized into PM&R, PC, and co-management categories. The table is intended to support integration into intake screening and referral workflows and to clarify service-level actions once a trigger is recognized.

The co-management triggers in Table 1 identify situations in which symptoms, function, and care planning are so closely linked that single-service management is likely to be incomplete. For example, when medication side effects such as sedation or decreased cognition create tradeoffs with functional ability, both services may need to coordinate safety planning. When care transitions coincide with declining mobility, PC may address goals of care and transition logistics while PM&R addresses caregiver transfer training, equipment needs, and rehabilitation goal alignment. In these situations, designating which service is responsible for specific clinical problems may be more effective than a general shared-care arrangement. The clinical vignettes in Section 4 illustrate these patterns in clinical context.

The table is also bidirectional to aid mutual referrals. Either specialty may recognize unmet needs that warrant involvement of the other, and co-management is frequently initiated because one service identifies a problem that falls within the other’s primary scope.

### 3.4. Primary-Service Triage Workflow (Figure 3)

This primary-service triage workflow was originally designed by reviewing the literature and integrating a neuro-oncology rehabilitation triage clinic tool [14] with palliative care referral criteria [18,19]. The tool was then reviewed and revised by cross-specialty clinicians for alignment.

This triage workflow is designed for clinical guidance rather than rigid implementation to decide which service is best positioned to be the primary supportive service or whether a combination of services would best address the patient’s current needs. The workflow begins with intake screening of symptoms, distress, function, and safety. Patient-reported outcomes that may be incorporated into this intake screening include the Edmonton Symptom Assessment System, NCCN Distress Thermometer, PROMIS Cancer Function Brief 3D Profile, and brief questions about falls, transfers, mobility, equipment needs, and caregiver strain [31,32,33,34]. This first step creates a shared set of signals that can be recognized across disciplines and reduces dependence on memory or individual referral habits.

**Figure 3:**
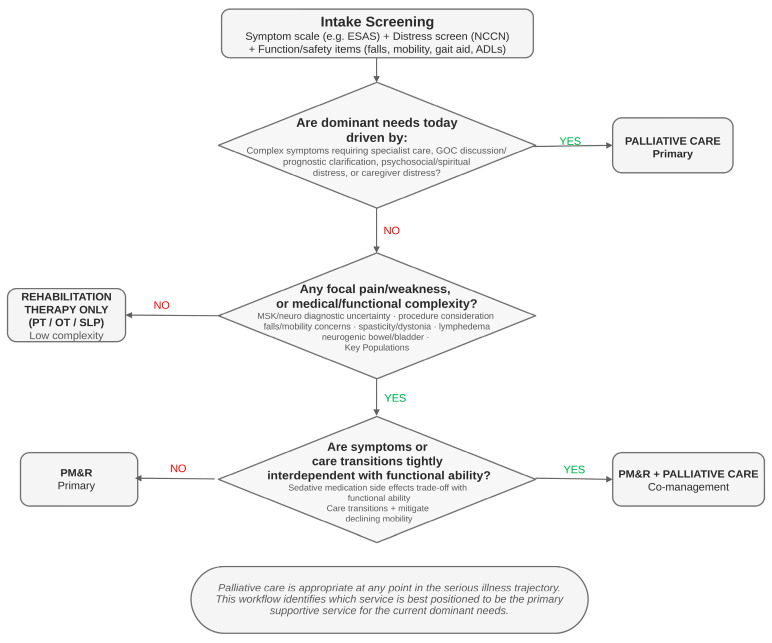
Primary-service triage workflow for referral to PM&R, PC, co-management, or rehabilitation therapy alone. The workflow is designed to identify which service should be the primary supportive service according to the patient’s current needs. PC may be appropriate at any point and is not restricted by this workflow. Arrows represent routing options rather than required routing sequence.

After intake screening, the first question is whether the dominant needs are driven by complex symptoms requiring specialist care, goals-of-care discussion or prognostic clarification, psychosocial or spiritual distress, or caregiver distress. If the answer is yes, PC should be the primary supportive service. This reflects the primary role of specialty PC in managing multidimensional suffering and care planning needs [6,7,18,19]. PC clinical needs are screened first as they are commonly urgent.

If those needs are not dominant, the second question is whether there is focal pain, weakness, mobility decline, or other medical or functional complexity that suggests a need for PM&R physician involvement. If the answer is no, the patient may be appropriate for rehabilitation therapy alone. This lower-complexity route is intended for patients whose needs can be addressed by PT, OT, or SLP without additional physician-level rehabilitation collaboration. This step was informed from clinical experience building a PM&R service line within a cancer center where referral decisions often centered on whether to refer to PM&R, directly to rehabilitation therapy, or both. These decisions can vary in different practice settings due to varying rehabilitation resources and patient-level factors. For example, PM&R can be the medical home for lymphedema with lymphedema therapy as the primary intervention. Depending on the access to and success of lymphedema screening and therapy, PM&R may not have to be involved.

If PM&R-relevant complexity is present, the third question is whether symptom burden or care transitions are tightly interdependent with functional ability. If they are, co-management between PM&R and PC should be the default. If they are not, PM&R should be the primary supportive service. PC may be appropriate at any point in the illness trajectory, including in parallel with PM&R or rehabilitation therapy alone.

## 4. Clinical Vignettes

Close collaboration between an oncology-specialized physiatrist and palliative care clinicians revealed substantial overlap in the patient populations served by both specialties. Many patients were receiving care from both services, often concurrently, but also often transitioning between services. This awareness highlighted the potential risks associated with fragmented coordination such as care inefficiencies with duplication of referrals, medication-related complications with shared symptom management, and inconsistent clinical messaging. These risks were mitigated by regular opportunities for joint evaluation and care planning.

We observed that these shared patients could be grouped into several recurring clinical patterns. Accordingly, the clinical vignettes presented in this article are hypothetical or derived from composite cases synthesized from multiple patients without identifiable patient data. These vignettes are intended to illustrate common trajectories encountered across the continuum of care and the ways in which patients may ultimately transition from, or no longer require, the integrated services described.

### 4.1. Case 1 (PM&R-Specific)—Stroke

A 50-year-old man with monoclonal gammopathy of undetermined clinical significance is evaluated 3 months after stroke with persistent weakness, gait impairment, dysphagia, and cognitive changes limiting independence and return to work. These ongoing functional deficits from a brain injury trigger a PM&R referral for goal-directed recovery and multidisciplinary rehabilitation coordination. Based on his deficits, outpatient multidisciplinary neurorehabilitation is recommended with expectations for gradual improvement over months. He was able to avoid aspiration pneumonia with swallow rehabilitation. His walking improves with PT, bracing, and botulinum toxin injections for spasticity. His cognition improves with neuropsychology and OT and he ultimately returns to office work with appropriate adaptations.

### 4.2. Case 2 (PM&R-Specific)—Post-Breast Surgery Shoulder Pain

A 40-year-old woman with history of early-stage breast cancer has persistent left shoulder pain after left mastectomy and reconstruction and adjuvant radiation treatment. Despite pain medications and doing her home exercises from PT daily for several months, her pain has progressed to severe, limiting use of her left arm. The focal pain refractory to PT triggers a PM&R referral for a diagnosis and injection evaluation. She is found to have no neuropathic symptoms and no concern for cancer recurrence. Bicipital tendonitis complicated by pectoralis dystonia from radiation fibrosis is diagnosed in the setting of persistent aggravating activity. Due to the severity, both steroid injection to the bicipital tendon and botulinum toxin injection to the pectoralis muscles are recommended. Also recommended are activity modification and timing PT visits soon after the procedure. The patient returns to unrestricted use of her arms.

### 4.3. Case 3 (PM&R-Specific)—Prehabilitation Before Rectal Cancer Surgery

A 70-year-old man with pancreatic cancer is scheduled for surgery in 4 weeks. His walking is stable and independent, but slow. He endorses feeling weaker overall over the last several years. The functional decline prior to major cancer surgery triggers a referral to PM&R for prehabilitation. PM&R prescribes a starting walking and strengthening home exercise program, refers to PT for further preoperative conditioning and postoperative movement strategies, discusses postoperative functional recovery, introduces protein intake recommendations, incorporates mental wellness strategies, and screens for multidisciplinary referral needs. The patient improves functionally prior to surgery, develops a sense of control, and understands early and safe post-operative mobilization.

### 4.4. Case 4 (PM&R-Specific)—Prehabilitation to Rehabilitation

A 50-year-old woman starting neoadjuvant chemotherapy for breast cancer is referred to PM&R for prehabilitation. Pre-existing neck pain is addressed with trigger point injections, PT, a tool for self-massage, and stress reduction strategies. A customized prehabilitation program is also provided as in Case 3. The neck pain improves; however, with rapid physical activity escalation, she develops greater trochanteric pain syndrome managed with a separate course of PT. Following surgery and adjuvant radiation treatment, she discontinues exercise and starts adjuvant chemotherapy. She is encouraged to restart exercising with further management of emerging cognitive changes and chemotherapy-induced peripheral neuropathy (CIPN) deprioritized. Subsequently on aromatase inhibitor therapy, the following issues are addressed: aromatase inhibitor-associated musculoskeletal symptoms, balance and dexterity issues from taxane-induced peripheral neuropathy, fatigue, cancer-related cognitive impairment, body image concerns, and sexual dysfunction. Care is sequenced according to patient and medical priorities including PT, cognitive rehabilitation, hand therapy, neuropsychology, exercise, pharmacologic symptom control, and acupuncture. When no further PM&R-specific needs are identified, she is counseled on potential future PM&R-specific needs such as lymphedema, post-breast surgery pain (Case 2), and anthracycline-induced cardiomyopathy prior to discharge.

### 4.5. Case 5 (Shared Needs → PM&R-Dominant → PC-Dominant → PC-Specific)—Cervical Dystonia

A 60-year-old man with metastatic lung cancer to bone presents for follow-up with PC for refractory neck pain and functional decline, triggering a referral to PM&R. PM&R determines the neck pain is from cervical dystonia secondary to chronic radiation fibrosis following treatment for head and neck cancer, without contributing etiologies such as myofascial pain, facetogenic pain, cervical radiculopathy, cervical spinal stenosis, or concern for disease progression. PM&R initiates botulinum toxin chemodenervation and targeted PT with improvements in pain and exercise tolerance. In shared management for concurrent back pain with multifactorial etiologies, PC manages pharmacological symptom control, while PM&R evaluates for musculoskeletal and neurologic etiologies and their injection options from different disciplines, and optimizes multimodal conservative care involving rehabilitation, spine hygiene, exercise safety, bracing as needed for daily activities, and acupuncture. Each service reinforces the other’s recommendations with clinical needs shifting to PM&R-dominant clinical needs in the setting of disease and symptom control over a few years.

While primarily under PM&R care, the patient develops progressive fatigue and dyspnea limiting exercise tolerance. He is found with disease and symptom progression, triggering urgent re-engagement with PC. Supportive care shifts to PC-dominant clinical needs with PM&R addressing functional expectations, caregiver training, anticipatory adaptive equipment, pressure injury prevention, and exercise modifications to mitigate progressive functional decline and loss of independence. Frequent hospitalizations and alterations in mental status with further disease progression limit outpatient engagement with rehabilitation interventions, and care transitions to PC-specific clinical needs.

### 4.6. Case 6 (PM&R-Dominant → Shared Needs → PC-Dominant)—CIPN Pain

An 80-year-old woman with diffuse large B-cell lymphoma is referred to PM&R for severe bilateral foot pain from CIPN limiting safe ambulation. PM&R initiates neuropathic pain medications, prescribes a gait aid, and provides supportive counseling for psychosocial and spiritual distress related to functional decline. With persistent pain and distress despite optimization of non-opioid pain management, PM&R refers to PC for complex symptom management and existential distress. In shared management, PC recommends low-dose methadone, as well as psychiatry and chaplaincy support, which PM&R reinforces. With improved pain, gait, and coping, the focus of PM&R care shifts toward functional goals, environmental safety, and encouragement of gait aid use. Subsequently, she develops marked fatigue requiring discontinuation of outpatient PT, prompting communication with the care team. Steroids are initiated for disease progression, with improvement in symptoms and mobility. Care then transitions primarily to PC in the absence of further modifiable PM&R-specific needs.

### 4.7. Case 7 (Rehabilitation Therapy Only)

A 65-year-old woman with metastatic breast cancer and cancer-related lymphedema in her arm. She previously completed a course of lymphedema therapy and has been managing well with mild worsening recently. Her last compression sleeve was obtained more than 6 months ago, and she recently moved from out-of-state and cannot contact the vendor to reorder. She is referred to lymphedema therapy directly. She is also encouraged to improve her exercise to reach guideline recommendations for aerobic and resistance exercise and does not anticipate any difficulty with accomplishing this. She has mild shoulder pain that she does not currently want to address. There are no other musculoskeletal or neurological issues impacting her functional ability.

### 4.8. Case 8 (Rehabilitation Therapy Only)

A 60-year-old man with advanced prostate cancer would like to address androgen deprivation therapy-related mild generalized joint pains and deconditioning. He is motivated to exercise and willing to participate with PT-guided conditioning as he is uncertain of safety if he were to try exercising on his own. He has no other functional concerns with potential musculoskeletal or neurological etiologies. He does not have pelvic floor dysfunction or cognitive concerns that he wants to address. A PT referral is provided.

## 5. Discussion

The main contribution of this manuscript is to provide a practical framework for applying the complementary roles of PM&R and PC in outpatient oncology care. The prior literature has established that these fields share an important focus on quality of life and may work well together [2]. However, routine oncology practice still lacks a clear and repeatable way to decide when to involve PM&R, PC, or both specialty services. The framework proposed here addresses that need through four linked tools that define clinical scope, depict clinical-needs gradients, connect referral triggers to expected clinical actions, and guide primary-service triage.

In clinical practice, crossover skills without rigid scope boundaries are often beneficial and necessary. As oncologic processes progress, medical and functional complexity tend to increase together while patient bandwidth to engage in more medical care decreases. Patients may not want other supportive clinicians despite the presence of a clinical need. PC clinicians may need to address some function and safety concerns (Case 5, Case 7, Case 8), encourage exercise, and address equipment needs. PC clinicians may also need to identify when pain may have a musculoskeletal or neurological etiology requiring specialty care from PM&R, as in Case 5. PM&R clinicians may need to address some symptoms and goals of care (Case 5, Case 6) to optimize functional ability and activity-based goals. An actual collaborative supportive oncology clinical practice may evolve from Figure 1’s clear scope boundaries to the complementary gradients in Figure 2 as mutual understanding among PM&R and PC clinicians develops.

### 5.1. Implementation Considerations

The clinical tools proposed are intended as point-of-care implementation and conversation aids rather than a rigid referral structure. Implementation can vary depending on patient complexity, specialist clinician access, individual specialty clinician skillsets, clinical support staff availability, and EHR and leadership support. The implementation considerations below are in order of increasing resources.

Without additional resources or workflows, we propose the steps below to begin PM&R and PC collaboration. First, individual PM&R and PC clinicians may collaborate by gaining a mutual understanding of each other’s skillsets via conversation, clinical shadowing, and study. For both PM&R and PC, institutional resources influence specialty service capabilities and the multidisciplinary team’s composition and clinical coherence. Developing a strong mutual understanding can facilitate successful bidirectional patient referrals.

Second, collaborate formally on patient care, communicating clearly and specifically to patients and via clinical documentation. Review the other specialty’s documentation routinely and follow up, as relevant, on each other’s plan of care. Problem-based role clarification should be documented. For example, if there is mutual understanding on neuropathic pain medication management between PM&R and PC, and if the patient will not get confused, PM&R may document increasing the dose of gabapentin during their visit and that the next titration will be done by PC with further medication-based symptom management per PC. Alternatively, PM&R may intentionally schedule a follow-up visit between PC follow-ups for the possibility of quicker collaborative titration in the outpatient setting. Third, communicate informally often to build trust and enhance patient care. This can be via curbsides to clarify appropriate referrals and management expectations, warm hand-offs, and humbly learning from each other.

The next tier of implementation involves triaging ad hoc. This can be done by the referring oncology clinician or nurse navigator. The referrer can use the tools from this paper to determine the configuration of specialty PM&R and/or PC clinical services needed, communicate the importance of these services, and explain what the patient may expect. However, depending on individual clinicians’ familiarity with PM&R and PC services, relevant clinical needs may be under-recognized. On the supportive clinical service end, PM&R and PC clinicians can similarly use these tools to triage incoming referrals to streamline coordinated and comprehensive supportive care upon referral.

As referrals increase, timely successful consultation to either PM&R and PC can become challenging, with both patient-level and operational factors contributing. Patients who often need both services, such as those with advanced cancer on chemotherapy with poor performance status, also have the least capacity to engage with healthcare and the greatest burden of time toxicity of time spent with healthcare [35]. More referrals can compound the very physical and emotional burden these services are intended to relieve. Operationally, more referrals can lead to higher appointment absence rates [36]. This mismatch between clinical intent and patient bandwidth can lead to both patient harm and operational inefficiencies. Thus, resource stewardship is a critical consideration for successful supportive oncology care with limited PM&R and PC resources. The tools in this framework may be used by referring clinicians to stage referrals according to the patient’s dominant needs and capacity to engage with more healthcare.

The addition of a dedicated supportive care nurse navigator can improve efficiency and access to supportive care interventions [37]. A nurse navigator can use the tools proposed in this paper to triage upon receipt of a referral to PM&R or PC. They can standardize clinical intake involving patient-reported outcome measures as in Figure 3. This would enhance the consistency of screening for supportive needs and enhance screening beyond standardized questions. There would be an educational opportunity to discuss with the patient regarding the appropriate specialty service or services recommended, help patients prepare for a productive consultation (e.g., bring caregiver, obtain records from outside orthopedic offices, track pain medication use and efficacy), and educate patients on consultation expectations. Importantly, there would be opportunity to clarify misconceptions about either specialty and to align the involvement of supportive services with patient capacity to help ensure that patients follow through with the consultation after referral. These processes reduce reliance on referring clinicians’ variable experience with supportive oncology specialties.

With system leadership investment, implementation can advance to routine clinical screening automatically triggered through the EHR at routine time intervals and linked to specific events such as changes in care setting (e.g., discharge from acute care, discharge from skilled nursing facility) and upon referral to either specialty. These screening results can then link to referral prompts with referral guidance tools such as those provided in this paper. A clinician can then refer to the appropriate service(s) and educate the patient on the reason for referral and the expectations for management.

### 5.2. Clinical Implications

This clinical framework may improve early referrals to both PM&R and PC. Early supportive oncology referral upon cancer diagnosis is a domain uniquely fit for PM&R involvement. Cancer rehabilitation services can be meaningful before, during, and after early cancer treatment, in addition to palliative settings [38]. However, although cancer treatments cause functional morbidity in most survivors, few receive rehabilitation services despite guideline-recommended care [16]. When rehabilitation services do get initiated, often it is after substantial functional decline when restoration is more difficult. In response, over the last few decades, attention has been shifting from a reactive, post-treatment cancer rehabilitation model to a proactive cancer prehabilitation model [39]. Cancer prehabilitation, optimizing physical and psychological health before cancer treatment, has become a global phenomenon with increasing literature showing not just better quality of life and functional abilities [40], but also improvement of cancer-related outcomes such as chemotherapy completion [41], pathologic complete response [42], and postoperative complications [43]. Subsequently, prospective surveillance and early rehabilitation can mitigate functional morbidity from impairments that may be cancer-related, cancer-treatment-related, or attributable to pre-existing or new comorbidities unrelated to cancer [39,44,45].

Early referrals to PC are recommended as part of high-quality oncology care and are recognized to improve quality of life in advanced cancer [6,46,47]. Even so, PC is often a late, crisis-driven service. PC referrals are often delayed by prognostic uncertainty, end-of-life stigma, concern that consultation will diminish hope [48,49], or contingency on failure of other services, where it is often appropriate earlier in the illness trajectory before a crisis [6,7,47]. In our clinical experience, PM&R is often involved earlier than PC in supportive cancer care for a patient. A PC-familiar PM&R physician could facilitate early PC referral for proactive, comprehensive supportive oncology care, thereby potentially reducing crisis-driven end-of-life care.

Increased referrals of the PC patient population to PM&R may result from this clinical framework. Function-directed rehabilitation interventions in the palliative oncology setting are meaningful [50]. However, rehabilitation referrals are often also deferred due to patient and clinician misperceptions of rehabilitation’s role and benefits. They may view rehabilitation as optional rather than essential and perceive limited benefit in progressive disease or in expecting that effective cancer treatment will reverse disablement [50,51]. Clinician-level barriers to cancer rehabilitation referrals include insufficient education, prognostic gatekeeping whereby oncologists withhold referrals from patients with limited life expectancy despite acknowledging rehabilitation’s value, and undervaluation of rehabilitation relative to disease-directed therapies [16,52,53,54,55]. PM&R and rehabilitation therapy services can remain meaningful in the palliative setting when goals shift from functional restoration to optimization [50,56,57,58,59]. In advanced progressive disease, the goal is to mitigate functional decline through the preservation of safe mobility, reduction in symptom interference with function, caregiver training, and adaptation to continue participation in meaningful activities [50,59] as illustrated in Cases 3 and 4. In palliative settings, the decision to involve rehabilitation services should remain needs-based rather than contingent on a specific prognostic threshold. A PM&R-familiar PC clinician could more-comprehensively address functional impairments.

### 5.3. Limitations

This clinical framework is intended for practice design and local testing, not as a prospectively validated decision instrument or formal guideline. Content review was limited to the co-authors and did not involve formal validation, structured review processes, or an external expert panel. The tools and clinical vignettes were developed from the collaborative clinical experience of a small PM&R and PC practice, including a PM&R physician subspecialized in cancer rehabilitation medicine with PC rotations in training, and PC clinicians with proclivities to recommend exercise and physical therapy. As crossover training is uncommon between the specialties of PM&R and PC, the proposed clinical framework was designed to facilitate collaboration in the context of limited pre-existing mutual understanding to improve replicability to other clinicians and oncology care settings.

## 6. Future Directions

Future work should focus on a phased evaluation for this framework moving from structured content validation to implementation and outcome evaluation according to implementation science models. This would allow for iterative refinement while generating evidence across measures of usability, process, and clinical impact before scaling to diverse oncology practice settings for broader adoption.

First, formal content validation should be undertaken both internally and externally. This may include a structured internal review process including the local expanding PM&R and PC clinical teams. Subsequently, structured review by an external multidisciplinary expert panel using established consensus methods such as the Delphi process or Nominal Group Technique could refine the clinical tools identifying consensus major criteria and adaptable minor criteria, supporting use across varying clinical contexts.

Second, implementation readiness in a clinical practice should be assessed with a formative evaluation of the framework’s acceptability, appropriateness, and feasibility among clinicians. This evaluation should be guided by Proctor et al.’s implementation taxonomy and the updated Consolidated Framework for Implementation Research (CFIR) user guide to predict implementation success [60,61]. The evaluation should involve multidisciplinary frontline clinicians across PM&R, PC, oncology, and other supportive oncology specialties. Using a mixed methods design combining structured surveys and semi-structured qualitative interviews (including think-aloud and cognitive interviewing techniques), investigators can assess perceived usability, workflow compatibility, and actionability. This process facilitates iterative refinement and stakeholder buy-in, thereby optimizing the framework’s contextual fit prior to pilot implementation.

Third, a prospective pilot implementation should evaluate the framework’s real-world performance using a hybrid Type 2 effectiveness-implementation design guided by the RE-AIM (Reach, Effectiveness, Adoption, Implementation, Maintenance) framework. A stepped-wedge cluster study design with practice sites adopting the framework in a staggered sequence would enable a pre–post comparison at each site, quantifying changes in both process and clinical effectiveness. This study design has previously been used for a trigger-based serious illness conversation intervention in oncology [22]. The implementation dimension should be evaluated by predefined process metrics that operationalize process reliability and implementation fidelity. These should include time from trigger recognition to referral initiation, trigger-to-service concordance, and the proportion of triggers resulting in completed consultations. Patterns of PM&R-PC co-management, including the frequency and directionality of referrals, should also be captured. Concurrently, clinical effectiveness outcomes should include functional status, time to functional decline, falls, symptom burden, and quality of life. Exploratory secondary outcomes where sample size permits may encompass crisis healthcare utilization and established end-of-life quality-of-care indicators endorsed by the National Quality Forum and the American Society of Clinical Oncology—including chemotherapy administration within 14 days of death, ICU admission in the last 30 days of life, and late or absent hospice referral [62]. Initial pilot clinical sites should be selected based on the presence of established PM&R and PC services, sufficient referral volume, and institutional capacity for prospective data capture.

Fourth, the above research phases should be evaluated across diverse oncology practice settings to assess the framework’s generalizability beyond well-resourced, larger oncology practice settings. This should include community oncology settings, safety-net institutions, and under-resourced systems. Where internal PM&R or PC services are unavailable, alternative care delivery models such as telerehabilitation, consultative partnerships, or regional referral networks should be explored.

Fifth, scaling the framework’s adoption would require investment in institutional and workforce resources. Regarding institutional resources, integration of the referral triggers into EHR clinical decision support systems is key for replicability and scalability. Automated alerts integrating PM&R and PC referral triggers—such as PM&R key diagnoses, functional status scores, symptom severity thresholds, or disease progression milestones—may reduce reliance on recognition by individual clinicians. Regarding workforce resources, substantial investment in increasing both the PM&R and PC workforce is particularly critical. Both workforces are insufficient to meet the current and growing demand [63]. However, the oncology-specialized PM&R workforce is particularly nascent relative to the PC workforce and the growing survivorship population’s rehabilitative needs [64]. To address this gap a multipronged strategy is urgent: (1) equipping PM&R clinicians with foundational palliative care competencies, particularly since access to specialty PC is often crisis-driven; (2) cultivating oncology and PC clinicians with primary PM&R skills; and (3) expanding oncology-specific training across the broader PM&R workforce. Collectively, these infrastructure investments will be essential to scale and sustain the implementation of this framework beyond initial pilot phases.

This phased research agenda provides a pathway from conceptual framework to scalable, evidence-based practice, advancing the integration of PM&R and PC across the continuum of oncology care.

## 7. Conclusions

Cancer care requires simultaneous attention to symptom burden, functional ability, and caregiver capacity. PM&R and specialty PC provide distinct but complementary value, and their overlap is often a key area for synergistic co-management. This paper presents four practical tools to support consistent bidirectional referral in routine oncology care, including a scope and overlap map, a clinical-needs gradient, a referral trigger table linking rationale and management expectations, and a primary-service triage workflow. As involvement of each specialty can vary along a gradient of dominant needs, this framework is designed to guide routing decisions within a fluid clinical landscape rather than to impose rigid specialty boundaries. Together, these tools and the provided clinical vignettes offer a framework for clarifying service roles, improving supportive care coordination in cancer care, and supporting earlier needs-based referrals. Future work should evaluate implementation and clinical usefulness of these tools across diverse oncology settings.

## Data Availability

No new data were created or analyzed in this study. Data sharing is not applicable to this article.
